# Carfilzomib, Lenalidomide, and Dexamethasone Followed by Salvage Autologous Stem Cell Transplant with or without Maintenance for Relapsed or Refractory Multiple Myeloma

**DOI:** 10.3390/cancers13184706

**Published:** 2021-09-20

**Authors:** Marc-Andrea Baertsch, Mathilde Fougereau, Thomas Hielscher, Sandra Sauer, Iris Breitkreutz, Karin Jordan, Carsten Müller-Tidow, Hartmut Goldschmidt, Marc-Steffen Raab, Jens Hillengass, Nicola Giesen

**Affiliations:** 1 Hematology, Oncology and Rheumatology, University Hospital Heidelberg, 69121 Heidelberg, Germany; 2 Division of Biostatistics, German Cancer Research Center, 69121 Heidelberg, Germany; 3 National Center for Tumor Diseases, Heidelberg University Hospital, 69121 Heidelberg, Germany; 4 Clinical Cooperation Unit Molecular Hematology/Oncology, German Cancer Research Center, 69121 Heidelberg, Germany; 5 Roswell Park Comprehensive Cancer Center, Buffalo, NY 14203, USA

**Keywords:** multiple myeloma, salvage high-dose chemotherapy, salvage autologous stem cell transplantation, lenalidomide maintenance

## Abstract

**Simple Summary:**

High-dose chemotherapy and autologous stem cell transplantation (HDCT/ASCT) is a standard treatment in patients with newly diagnosed multiple myeloma (MM). At relapse, salvage HDCT/ASCT is a treatment option in patients with sufficient benefit from frontline HDCT/ASCT, but no evidence is currently available regarding its role in the era of triplet regimens combining the most active drug classes for relapsed MM. To evaluate the outcome after salvage HDCT/ASCT following re-induction treatment with carfilzomib/lenalidomide/dexamethasone (KRD) and to identify prognostic factors, we conducted a retrospective analysis of patients that had previously undergone frontline HDCT/ASCT. We found that deep remissions achieved with KRd followed by salvage autologous transplantation were associated with favorable PFS and were enhanced by maintenance treatment. Salvage autologous transplantation after state-of-the-art triplet re-induction was a safe and effective strategy for RRMM patients that may offer the chance to avoid refractoriness to multiple novel agents at the next relapse.

**Abstract:**

Salvage high-dose chemotherapy and autologous stem cell transplantation (HDCT/ASCT) is a treatment option for relapsed and/or refractory multiple myeloma (RRMM). No data are available on salvage HDCT/ASCT following re-induction treatment with state-of-the-art triplet regimens. We retrospectively report on 44 patients receiving salvage HDCT/ASCT following re-induction with carfilzomib/lenalidomide/dexamethasone (KRd). All patients received frontline HDCT/ASCT with median time to progression (TTP1) of 2.9 (1.2–13.5) years, enabling paired comparison of frontline and salvage HDCT/ASCT. After re-induction and before salvage transplant, 25/44 patients (57%) attained ≥ very good partial response (VGPR), which increased to 34/44 (77%) at best response after salvage HDCT/ASCT. Median progression-free survival (PFS) was 23.3 months from salvage HDCT/ASCT. Patients with ≥ VGPR at the time of salvage HDCT/ASCT and those receiving maintenance treatment post salvage HDCT/ASCT had significantly superior PFS (hazard ratio (HR) 0.19, *p* = 0.001 and HR 0.20, *p* = 0.009). In patients achieving at least an equal depth of response before salvage HDCT/ASCT as before frontline HDCT/ASCT, PFS after salvage HDCT/ASCT was comparable to the frontline situation (*p* = 0.3). This is the first report of state-of-the-art triplet re-induction and salvage HDCT/ASCT for RRMM after frontline transplantation. Deep remissions achieved with KRd translate into prolonged PFS following salvage HDCT/ASCT and are enhanced by maintenance treatment.

## 1. Introduction

Despite significantly improved prognosis of multiple myeloma (MM) following the approval of novel agents, including several proteasome inhibitors (PI), immunomodulators (IMiD), and monoclonal antibodies during the past decade, MM remains an incurable malignancy. The course of symptomatic MM is characterized by a succession of treatment-induced remissions and relapses that ultimately result in multi-refractory disease with poor prognosis [1].

The role of high-dose chemotherapy with melphalan followed by autologous stem cell transplantation (HDCT/ASCT) in frontline treatment of MM is well established and remains the standard of care for eligible patients even in the era of novel agents [2,3,4]. At relapse, multiple treatment options are available, including salvage transplant and non-transplant strategies. IMID (lenalidomide (LEN))- and/or PI (bortezomib (BTZ), carfilzomib (CFZ))-based triplet regimens can induce deep, durable remissions in many patients and have become a treatment standard in recent years [5,6,7,8,9]. Salvage HDCT/ASCT is used in clinical practice with the intent to deepen and prolong remissions. However, increased potential for HDCT/ASCT-associated toxicity in RRMM patients remains a concern. The use of salvage HDCT/ASCT is supported by a randomized controlled phase III trial (NCRI Myeloma X Relapse) that demonstrated improved PFS (19 vs. 11 months) and OS (67 vs. 52 months) compared to conventional dose cyclophosphamide consolidation for 12 weeks [10,11]. However, evidence for its benefit in the era of novel agents is limited [12]. The only randomized controlled phase III trial (GMMG ReLApsE) that compared a novel agent regimen including salvage HDCT/ASCT versus continuous novel agent treatment (LEN/dexamethasone[DEX]) failed to show a PFS (21 vs. 19 months) or OS (not reached vs. 63 months) benefit in the primary analysis but was hampered by a ~ 30% discontinuation rate before the transplant step [13]. Post-hoc landmark analyses of this trial suggested that some benefit was retained in patients that actually received salvage HDCT/ASCT. Currently, no data are available regarding the effect of salvage HDCT/ASCT after frontline transplantation in the context of re-induction with state-of-the-art triplet regimens.

The present analysis therefore aims to investigate the outcome after CFZ/LEN/DEX (KRd) re-induction followed by salvage HDCT/ASCT with or without post-transplant maintenance treatment and to evaluate the prognostic value of each patient’s individual outcome parameters from frontline HDCT/ASCT for the salvage transplant setting.

## 2. Patients and Methods

### 2.1. Patients

We identified *n* = 44 patients who received salvage HDCT/ASCT after re-induction treatment with KRd between April 2016 and April 2018 at our institution, a tertiary referral center, including 3 patients (7%) in whom LEN was primarily omitted due to intolerance (*n* = 2) or refractoriness (*n* = 1). All patients had received frontline HDCT/ASCT and had progressive disease according to IMWG definition [14] at the time of re-induction initiation.

### 2.2. Assessments

Baseline characteristics, outcome, and safety parameters were extracted by retrospective chart review. Comorbidities were assessed at diagnosis and at relapse according to the score published by Sorror et al. [15]. International Staging System (ISS) stages were calculated from albumin and beta-2-microglobulin levels at diagnosis [16]. Recurrent cytogenetic aberrations as identified by interphase fluorescence hybridization (iFISH) at diagnosis were grouped according to previously published standard and high-risk categories [17]. Cytogenetic high-risk included deletion17p, translocation 4;14, translocation 14;16, and gain1q (>3 copies); all other aberrations were considered standard risk. Treatment response and progressive disease were assessed according to IMWG criteria [14] complemented by minimal response (MR) according to EBMT criteria and near complete remission (nCR) [18,19]. Overall response rate (ORR) was defined as partial response (PR) or better. Response status post HDCT/ASCT was determined at the next follow up visit to our outpatient clinic (~2 months after HDCT/ASCT). Best response refers to the best response level achieved at any time post-transplant and before progressive disease. Survival was assessed from the time of ASCT until progressive disease (time to progression (TTP)), progressive disease or death (PFS), and death (OS).

Antibiotics were classified as prophylaxis (oral ciprofloxacin or daily cotrimoxazole), broad-spectrum beta-lactams with coverage of pseudomonas aeruginosa (piperacillin/tazobactam, ceftazidime), carbapenems (meropenem, imipenem), antibiotics with selective coverage of gram-positive bacteria (vancomycin, linezolid, and daptomycin), and reserve antibiotics. Mucositis was graded according to world health organization (WHO) [20]. A scale from 0 (no pain) to 10 (worst pain imaginable) was used to grade pain. Sepsis was defined as the presence of systemic inflammatory response syndrome (SIRS) plus an infectious focus.

### 2.3. Treatment

Patients received KRd as re-induction treatment at the standard schedule and dose published in the ASPIRE trial [5]. CFZ was administered intravenously on days 1, 2, 8, 9, 15, and 16 (starting dose 20 mg/m^2^ on days 1 and 2 of cycle 1; target dose 27 mg mg/m^2^ thereafter); LEN 25 mg was given orally on days 1–21; and DEX 40 mg was given on days 1, 8, 15, and 22 of 28-day cycles. Doses could be reduced at the discretion of the treating physician. HDCT consisted of melphalan 200 mg/m^2^ (100 mg^2^ intravenously on days −2 and −3). For ASCT, ≥2*10^6^ CD34^+^ cells per kg bodyweight were infused on day 0; excess stem cells were collected during frontline treatment and cryopreserved for use during salvage transplantation. Patients were either admitted for inpatient care or visited our outpatient clinic daily for HDCT/ASCT and subsequent bone marrow aplasia at least until reconstitution of peripheral blood neutrophils >0.5/nL or leucocytes >1/nL and platelets >20/nL. Maintenance treatment after salvage HDCT/ASCT was given at the discretion of the treating physician.

### 2.4. Statistics

Paired statistical tests were used to compare variables between frontline and salvage HDCT/ASCT in the same patients. Continuous variables were tested for differences between frontline and salvage HDCT/ASCT by Wilcoxon signed rank test. Dichotomous, categorical variables were compared by McNemar’s test and non-dichotomous, categorical variables by Stuart Maxwell’s test. Fisher’s exact test was used to compare unpaired dichotomous variables. PFS, time to progression (TTP), OS, and time to reconstitution of peripheral blood counts were all calculated from ASCT according to Kaplan–Meier and compared between groups by logrank test (unpaired data) or stratified log-rank test using individual patients as strata (paired data). Univariate and multivariate Cox regression analyses were applied to identify prognostic factors for PFS after salvage HDCT/ASCT. Maintenance treatment after salvage HDCT/ASCT was considered as a time-dependent covariate, and PFS according to maintenance was depicted as Simon–Makuch plot. Binary logistic regression analysis was performed to assess the repetition risk of adverse events and association of depth of response between frontline and salvage treatment. All statistical analyses are exploratory and were performed in SPSS (v24 and v26, IBM), Prism (v6, GraphPad), and R studio. A significance level α of 0.05 was used.

## 3. Results

Patient characteristics are shown in Table 1. Median TTP after frontline HDCT/ASCT (TTP1) was 2.9 (range 1.2–13.5) years. Induction treatment before frontline HDCT/ASCT consisted of bortezomib (BTZ)-based triplets in 38/44 patients (86%). The median number of prior lines of therapy at the time of re-induction was 1 (range 1–3). At the time of re-induction, 16/44 patients (36%) had previous LEN exposure and all patients were CFZ-naïve. High-risk features according to cytogenetics and ISS III were present in 10/37 (27%) and 10/42 (24%), respectively, at the time of diagnosis. Patients scored higher on the Sorror comorbidity index at the time of relapse compared to the frontline situation (*p* = 0.002) due to occurrence of heart disease (*n* = 12), liver disease (*n* = 4), pulmonary disease (*n* = 4), psychiatric disease (*n* = 4), and second primary malignancy (*n* = 1) in the meantime.

Patients received a median of 3 (range 3–9) cycles of re-induction treatment. Salvage HDCT/ASCT consisted of melphalan 200 mg/m^2^ in 41/44 patients (93%) and was reduced to 100–140 mg/m^2^ in 3/44 patients (7%; 1 each due to renal insufficiency, chronic heart failure and impaired general condition). Maintenance treatment post salvage HDCT/ASCT was given in 17/44 patients (39%), most frequently LEN 10–15 mg/d (16/44; 36%).

### 3.1. Response and Survival

**Figure 1:**
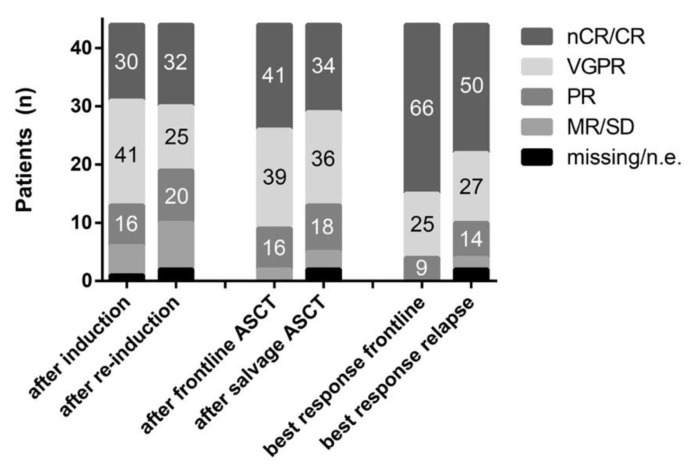
Depth of response after (re-)induction, frontline/salvage HDCT/ASCT, and at best response. Numbers on bars are percentages. *CR*: complete remission; *nCR*: near CR; *VGPR*: very good partial response; *PR*: partial response; *MR/SD*: minimal response/stable disease; and *n.e.*: not evaluable.

After re-induction treatment ORR, ≥VGPR and ≥nCR rates were 77% (34/44), 57% (25/44), and 32% (14/44), respectively. Responses deepened to 89% (39/44), 70% (31/44), and 34% (15/44) after salvage HDCT/ASCT and 90% (40/44), 77% (34/44), and 50% (22/44) at best response (Figure 1). Depth of response did not differ significantly between salvage and frontline treatment at the corresponding timepoints “after (re-)induction” (ORR *p* = 0.29, ≥VGPR *p* = 0.15, ≥nCR *p* = 1.0), “after (salvage) HDCT/ASCT” (ORR *p* = 1.0, ≥VGPR *p* = 0.58, ≥nCR *p* = 0.77), and “best response” (ORR *p* = 0.5, ≥VGPR *p* = 0.13, ≥nCR *p* = 0.18). Patients achieving deep remissions during frontline treatment were more likely to re-achieve deep remissions in the salvage setting at each individual timepoint: after (re-)induction (≥VGPR rate: odds ratio (OR) 6.22; 95% confidence interval (95%-CI) 1.33–29.01; *p* = 0.02), after (salvage) HDCT/ASCT (≥nCR rate: OR 5.71; 95%-CI 1.44–22.62; *p* = 0.01), and at best response (≥nCR rate: OR 4.50; 95%-CI 1.12–18.13; *p* = 0.03).

**Figure 2:**
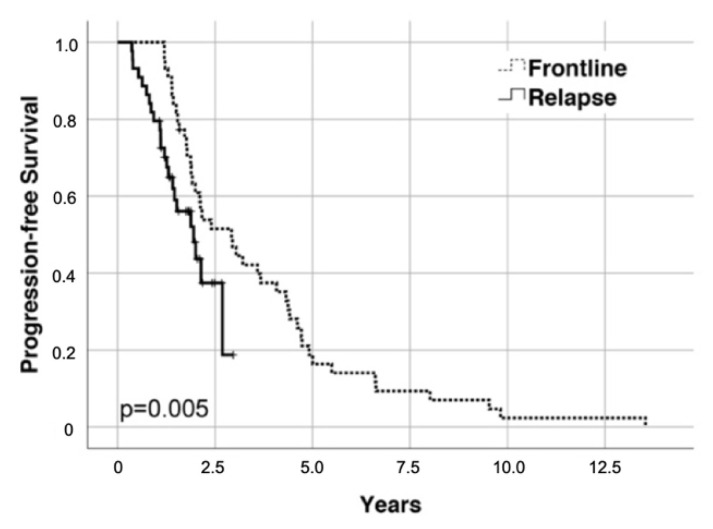
Kaplan–Meier plot of progression-free survival (PFS) from frontline and salvage ASCT, respectively.

**Figure 3:**
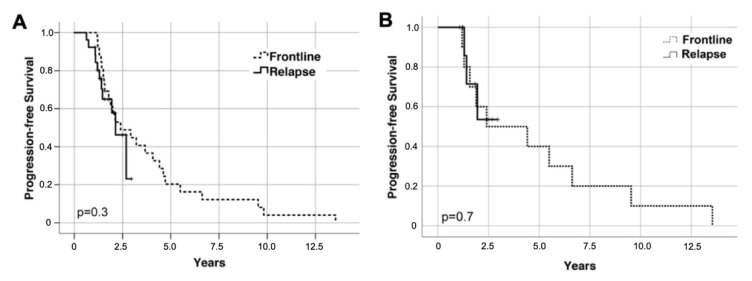
Kaplan–Meier plots of progression-free survival (PFS) in patients (**A**) achieving at least equal and (**B**) better depth of response at the time of salvage HDCT/ASCT as before frontline HDCT/ASCT PFS was calculated from ASCT.

After a median follow up of 23.9 months after salvage ASCT, 23 PFS and 3 OS events occurred. Median PFS from salvage ASCT was 23.3 months (Figure 2), and median OS was not reached (27.4 months and not reached, respectively, when calculated from initiation of re-induction treatment). Median PFS from HDCT/ASCT at relapse was significantly shorter than after frontline HDCT/ASCT (35.0 months; *p* = 0.005) in the overall cohort. Patients achieving at least an equally deep remission (*n* = 26/44 (59%)) or a deeper remission (*n* = 10/44 (23%)) at the time of salvage HDCT/ASCT as compared to frontline HDCT/ASCT had similar PFS in both lines of treatment (*p* = 0.3 and *p* = 0.7; Figure 3).

### 3.2. Prognostic Factors

**Figure 4:**
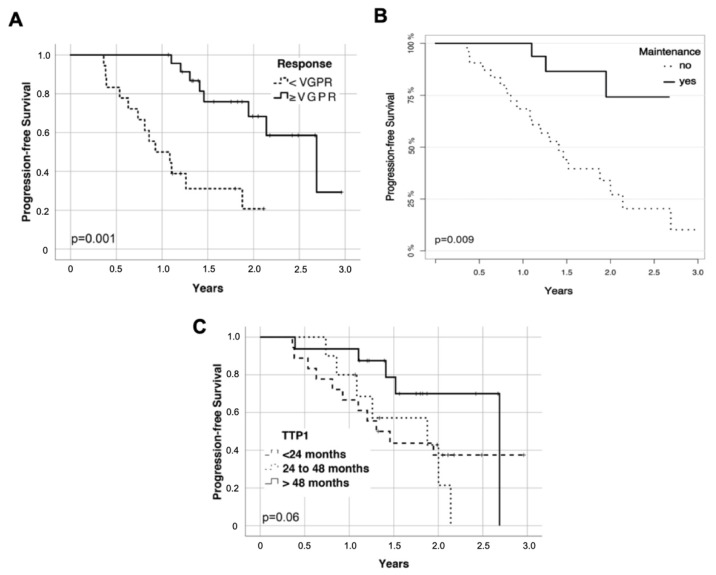
Progression-free survival (PFS) according to (**A**), Kaplan–Meier plot response at the time of salvage HDCT/ASCT, (**B**), Simon–Makuch plot maintenance treatment after salvage HDCT/ASCT, and (**C**), Kaplan–Meier plot time to progression after frontline HDCT/ASCT (TTP1) PFS was calculated from salvage ASCT.

Univariate analysis (Table 2) of potential prognostic factors revealed significant associations of response status at the time of salvage HDCT/ASCT (≥VGPR; hazard ratio (HR) 0.19; *p* = 0.001; Figure 4A) and maintenance treatment after salvage HDCT/ASCT (HR 0.20; *p* = 0.009; Figure 4B) with superior PFS after salvage HDCT/ASCT. The number of prior lines of therapy (>1; HR 5.7, *p* = 0.001) and prior lenalidomide exposure (HR 2.68; *p* = 0.02) were associated with inferior PFS after salvage HDCT/ASCT. A trend towards superior PFS after salvage HDCT/ASCT in patients with longer TTP1 (HR 0.79, *p* = 0.06; Figure 4C) was observed. No significant associations with PFS after salvage HDCT/ASCT were observed for age, or maintenance treatment, after frontline HDCT/ASCT. Response status after frontline HDCT/ASCT or at best response during frontline treatment were also not associated with PFS after salvage HDCT/ASCT. Kaplan–Meier plots of PFS after frontline and salvage HDCT/ASCT according to response are shown in supplementary Figure S1.

On multivariate analysis (Table 3), response status at the time of salvage HDCT/ASCT (≥VGPR; HR 0.18; *p* = 0.001) and maintenance treatment after salvage HDCT/ASCT (HR 0.22; *p* = 0.02) retained statistically significant associations with superior PFS after salvage HDCT/ASCT. Prior lenalidomide exposure and the number of prior lines of therapy lost their significance.

### 3.3. Safety

**Figure 5:**
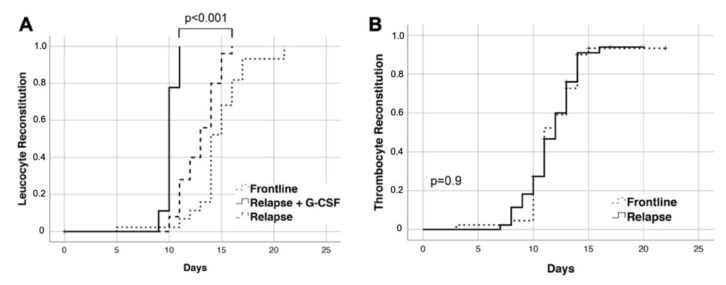
Time to reconstitution of peripheral blood counts. (**A**) Time to reconstitution of peripheral blood leukocytes (>1/nL) according to frontline and salvage HDCT/ASCT. For salvage HDCT/ASCT, reconstitution with and without G-CSF use was plotted separately. (**B**) Time to reconstitution of peripheral blood platelets (>20/nL) according to salvage and frontline HDCT/ASCT. Time to reconstitution was calculated from ASCT.

Median duration of the hospital stay was similar between frontline and salvage HDCT/ASCT (21 and 20 days). Median time to reconstitution of peripheral blood leukocytes (>1/nL) was shorter after salvage compared to frontline HDCT/ASCT (11 vs. 14 days; *p* < 0.001). After salvage but not after frontline HDCT/ASCT, 18/44 patients (41%) received G-CSF until leukocyte reconstitution, which was associated with faster reconstitution compared to patients not receiving G-CSF (*p* < 0.001; Figure 5A). Median time to platelet reconstitution did not differ between frontline and salvage HDCT/ASCT (12 vs. 11 days; *p* = 0.9; Figure 5B).

An overview of adverse events, antimicrobial management, and transfusions during the transplant phase is given in supplementary Table S1. Fever of unknown origin (FUO) constituted the most frequent infectious adverse event (32/43 (74%) vs. 29/44 (66%); *p* = 0.45). Bacteremia occurred in 10/41 (24%) and 6/39 patients (15%) in whom blood cultures were drawn (*p* = 0.39). Use of G-CSF had no significant effect on the frequency of FUO as the most common infectious adverse event (G-CSF 10/18 (56%) vs. no G-CSF 19/26 (73%); *p* = 0.33) or other infections. Two of 44 patients after salvage HDCT/ASCT were admitted to the intensive care unit (ICU) for mechanical ventilation (*n* = 1) and intravenous catecholamines (*n* = 1); no ICU admissions were required after frontline HDCT/ASCT. No transplant-related mortality occurred.

Oral antibiotic prophylaxis was given in 20/43 (47%) and 14/44 patients (32%) after frontline and salvage HDCT/ASCT, respectively. Median duration of i.v. antibiotic treatment was 8 days in both cases (*p* = 0.69). More broad-spectrum beta-lactam antibiotics with coverage of pseudomonas aeruginosa were given in patients after frontline (39/43 (91%)) compared to salvage HDCT/ASCT (32/44 (73%), *p* = 0.02). However, the use of broad spectrum i.v. antibiotics (beta-lactam antibiotics with coverage of pseudomonas aeruginosa and/or carbapenems) was not significantly different after frontline and salvage HDCT/ASCT (39/43 (91%) vs. 34/44 (77%), *p* = 0.11). More patients received erythrocyte transfusions during frontline compared to salvage HDCT/ASCT (18/44 (41%) vs. 12/44 (27%); *p* = 0.04).

Patients with mucositis of grade 2 or more after frontline HDCT/ASCT were more likely to re-develop at least grade 2 mucositis after salvage HDCT/ASCT (OR 5.13; 95%-CI 1.19–22-1; *p* = 0.03). No significant association of other adverse events after frontline HDCT/ASCT with re-occurrence after salvage HDCT/ASCT was observed, including FUO, bacteremia, sepsis, carbapenem use, selectively gram-positive antibiotics use, erythrocyte, or platelet transfusions.

## 4. Discussion

This is the first report on salvage HDCT/ASCT after state-of-the-art triplet novel agent re-induction in patients with RRMM after frontline transplantation. Our data show that deep and durable remissions can be achieved and suggest that maintenance treatment post salvage HDCT/ASCT is associated with favorable PFS. Despite increased comorbidity in the relapsed setting, salvage HSCT/ASCT can be performed safely in this patient population.

Successfully bringing patients to salvage HDCT/ASCT is challenging due to increased refractoriness of the disease and susceptibility of patients for severe adverse events compared to the frontline setting. In the GMMG phase III ReLApsE trial [13], ~30% of patients failed to receive the assigned salvage HDCT/ASCT, which was attributable to early disease progression in 12%. Similarly, in the NCRI phase III Myeloma X Relapse trial, 41% of registered patients did not reach the post re-induction (PAD) randomization stage, including 10% of patients who progressed before the transplant stage of the trial [10]. While we are not able to assess rates of early disease progression with KRd re-induction due to selection of patients based on salvage HDCT/ASCT, we observed deep responses (≥VGPR) after KRd re-induction in the majority of patients (57%) that correlated with improved PFS after salvage HDCT/ASCT. This compares favorably with the ≥VGPR rate of 24% after LEN/DEX re-induction in patients that received salvage HDCT/ASCT in the GMMG ReLApse trial [13] and is in line with findings from the phase III ASPIRE trial that showed more rapid induction of deeper responses with KRd vs. LEN/DEX [5].

The paired comparison with frontline HDCT/ASCT was chosen to evaluate the prognostic value of a patient’s previous outcome parameters in the setting of salvage HDCT/ASCT. Patients that attained at least an equally deep response before salvage HDCT/ASCT as in the frontline setting achieved comparable PFS. This information may be valuable to roughly gauge the PFS outcome of salvage HDCT/ASCT after KRD in the individual patient and underlines the importance of a potent re-induction regimen. Conversely, depth of response during frontline HDCT/ASCT was not directly associated with PFS after salvage HDCT/ASCT and thus does not qualify as a parameter for selection of patients for salvage HDCT/ASCT based on the present data.

Consistent with the literature [21], we observed a trend towards superior PFS after salvage HDCT/ASCT in patients with longer TTP1 after frontline HDCT/ASCT. Importantly, the impact of TTP1 on PFS after salvage HDCT/ASCT in our analysis was less pronounced than that of depth of response and maintenance in the context of salvage HDCT/ASCT. This suggests that a shorter TTP1 within the limits stated above may be compensated by effective re-induction and maintenance treatment to some degree. Typically, TTP1 of 12–18 months has been regarded as a minimum requirement for salvage HDCT/ASCT; however, with now standard use of maintenance after frontline treatment, higher cutoffs are suggested [21]. In this regard, the absence of a negative effect of maintenance after frontline HDCT/ASCT on PFS after salvage HDCT/ASCT in our cohort is encouraging and confirms an earlier report [22]. Considering the limited size of our cohort and thus limited statistical power, undertreatment in light of today’s standard for frontline treatment (especially LEN maintenance) likely still contributed to the positive outcomes observed at relapse.

Despite achieving deep responses with KRd re-induction treatment that further deepened with salvage HDCT/ASCT, a similarly important prognostic factor for PFS in our analysis was maintenance treatment after salvage HDCT/ASCT. This is in line with the frontline setting where LEN maintenance is standard of care based on PFS and OS benefit [2,3,23].

With the most effective continuous triplet regimens, median PFS is now between 26 (KRd; ASPIRE trial) [24] and 45 months (Daratumumab/LEN/DEX [DRd]; POLLUX trial) [25] and PFS at 2 years is between ~55% and ~75%, respectively, in less heavily pretreated RRMM patients (median 1–2 prior lines of therapy). Median PFS from salvage HDCT/ASCT in our overall cohort was 23 months (27 months from initiation of re-induction treatment) and was not reached in the subgroup with maintenance. The latter had PFS of ~75% at 2 years and compares favorably with patients that received salvage HDCT/ASCT and maintenance following LEN/DEX reinduction in the GMMG ReLApsE trial [13] (median PFS 23 months from salvage HDCT/ASCT). It is important to note that our cohort is biased by selection based on minimum TTP1 of 12 months and eligibility for salvage HDCT/ASCT after re-induction—both factors that likely select for good prognosis and that are not regularly assessed/reported outside of the transplant setting, thus limiting comparability with published triplet regimen trials. Moreover, due to inclusion at the stage of salvage HDCT/ASCT, our cohort does not capture patients who may have had early disease progression during the first KRd cycles and went on to other salvage treatments. Compared to the KRd arm of theASPIRE trial [24], our patients have fewer prior lines of treatment (median 1 vs. 2) and are younger (median age 59 vs. 64 years). Other characteristics such as WHO performance score (0-I in 84% vs. 90%) and renal function (creatinine < 2 mg/dl in 98% vs. creatinine clearance > 50 mL/min in 93%) are similar. A sub-analysis from the ASPIRE trial focused on patients with TTP1 of at least 12 months after frontline HDCT/ASCT who received KRd as second-line treatment reported PFS of ~65% at 24 months and median PFS of 33.5 months [26]. From POLLUX, such detailed sub-analyses based on prior treatment are not published; in the overall DRd arm [25], the median number of prior treatment lines was 1, median age was 65 years, WHO PS was 0 in 49% and I-II in 51%, and median time from diagnosis was 3.5 years (median time from frontline transplantation in our cohort 2.9 years); the subgroup of patients with only one prior line of therapy in POLLUX achieved PFS of ~75% at 2 years [25]. However, due to the limitations stated above, these comparisons need to be interpreted very cautiously. A benefit of proceeding to salvage HDCT/ASCT after a certain number of re-induction cycles may lie in limiting exposure to combination therapy and reducing treatment intensity after the transplant step which may avoid refractoriness to multiple classes of novel agents and associated poor prognosis [27], although this is beyond the scope of the current analysis.

Our results are not directly applicable to the increasing number of patients with LEN refractoriness at early stages of the treatment course due to its routine use during frontline treatment. However, we assume that achievement of a deep remission at and administration of maintenance after salvage HDCT/ASCT will also be beneficial in the LEN refractory setting. Potent triple combinations without LEN, such as CFZ/daratumumab/DEX and daratumumab/BTZ/DEX, are now available and induce rates of VGPR or better that are comparable to those achieved with KRd (69%, 59%, and 69% according to CANDOR [28], CASTOR [8], and ASPIRE [5] trials, respectively). Moreover, efficacy of lenalidomide-free maintenance using CFZ/DEX has been demonstrated in the salvage HDCT/ASCT setting [29]. Such regimens represent attractive backbones in the context of salvage HDCT/ASCT that require further evaluation, especially since outcomes with standard treatments are consistently inferior for LEN refractory patients.

Limitations of our analysis besides its retrospective nature and the small cohort size are the lack of cytogenetic and ISS data at relapse. Both cytogenetic risk status and ISS stage at diagnosis did not significantly impact PFS from relapse (data not shown). Unfortunately, outside of clinical trials these parameters are not routinely collected at relapse, which precluded inclusion in our multivariate model. However, this may be partially compensated for by inclusion of TTP1, which can be viewed as an integrated marker of risk as it represents the behavior of the disease under prior treatment [26,30].

## 5. Conclusions

In conclusion, our data show that salvage HDCT/ASCT after state-of-the-art triplet re-induction is safe and results in deep and durable remissions in patients with RRMM. Furthermore, it may offer the chance to avoid refractoriness to multiple novel agents at the next relapse. Maintenance treatment was required to achieve PFS in the range of what is achieved with current triplet regimens administered until progression although comparability between studies is limited by selection bias in our cohort. Prospective, randomized controlled trials are needed to clarify the role of salvage HDCT/ASCT in today’s treatment landscape. However, in the absence of such data our analysis can support salvage HDCT/ASCT as an option in combination with triplet regimens and maintenance treatment in patients with sufficient benefit from frontline HDCT/ASCT.

## Figures and Tables

**Table 1:** Patient characteristics data are *n*/*n* tested (%) or median (range).

	Frontline Transplant	Salvage Transplant
Age (median (range))	54.8 (36–68)	58.9 (40–71)
Sex		
Female	22 (50%)	
Male	22 (50%)	
WHO PS		
0	15/44 (34%)	-
1	22/44 (50%)	-
2	7/44 (16%)	-
Sorror comorbidity score		
0	23/44 (52%)	11/44 (25%)
1	5/44 (11%)	8/44 (18%)
2	8/44 (18%)	10/44 (23%)
3	7/44 (16%)	9/44 (21%)
≥4	1 (2%)	6/44 (14%)
Myeloma subtype		
IgG	17/44 (39%)	
IgA	11/44 (25%)	
IgD	1/44 (2%)	
Bence Jones	8/44 (18%)	
Hyposecretory	7/44 (16%)	
Light chain subtype		
Kappa	29/44 (66%)	
Lambda	15/44 (34%)	
ISS		
I	23/42 (55%)	-
II	9/42 (21%)	-
III	10/42 (24%)	-
Cytogenetics		
Standard risk	27/37 (73%)	-
High risk	10/37 (27%)	-
Serum creatinine		
≤2	35/44 (80%)	42/43 (98%)
>2	9/44 (20%)	1/43 (2%)
LDH		
Normal	41/42 (98%)	38/42 (90%)
Elevated	1/42 (2%)	4/42 (10%)
Prior lines of treatment		
1	-	38/44 (86%)
2	-	3/44 (7%)
3	-	3/44 (7%)
(Re-)Induction treatment		
PAD	20/44 (46%)	-
VCD	18/44 (41%)	-
VAD	4/44 (9%)	-
Other	2/44 (5%)	-
KRD	-	41/44 (93%) */^&^
KD	-	3/44 (7%)
Cycles of (Re-)Induction		
3	34/44 (77%)	38/44 (86%)
4	8/44 (18%)	5/44 (11%)
6	2/44 (5%)	-
9	-	1/44 (2%)
HD-Melphalan		
200 mg/m^2^	41/44 (93%)	41/44 (93%)
140 mg/m^2^	-	2/44 (5%)
100 mg/m^2^	3/44 (7%)	1/44 (2%)
Single vs. tandem transplant		
Single	28/44 (64%)	44/44 (100%)
Tandem	16/44 (36%)	-
Maintenance treatment	22/44 (50%)	17/44 (39%)
Lenalidomide	8/44 (18%)	16/44 (36%) ^§^
Thalidomide	7/44 (16%)	-
Bortezomib	5/44 (11%)	1/44 (2%)
Interferon	2/44 (5%)	-
Lenalidomide pretreatment	-	16/44 (36%)

* One patient received an additional cycle of pomalidomide/cisplatin/doxorubicin/cyclophosphamide/etoposid (POM-PACE) due to suspected PD on imaging, which was not confirmed later on. ^&^ One patient each reduced to Kd and Rd due to exanthema and transaminitis on KRd treatment, respectively. ^§^ Three patients received additional dexamethasone for maintenance. *HD*: high-dose; *ISS:* international staging system; LDH: lactate dehydrogenase; *PAD*: bortezomib/doxorubicin/dexamethasone; *VAD*: vincristine/doxorubicin/dexamethasone; *VCD*: bortezomib/cyclophosphamide/dexamethasone; *WHO PS*: World Health Organization performance score.

**Table 2:** Univariate Cox regression analysis of prognostic impact on progression-free survival (PFS) from salvage HDCT/ASCT.

Variable	*n*	HR	95% CI	*p*
Age	44	1.01	0.95–1.06	0.83
Prior lines of therapy (>1 vs. 1)	**6/44**	**5.70**	**2.04–15.90**	**0.001**
TTP1 (per 1 year increase)	44	0.79	0.62–1.01	0.06
Response status at time of salvage transplant (≥VGPR vs. <VGPR)	**24/44**	**0.19**	**0.07–0.49**	**0.001**
Maintenance (salvage transplant)	**17/44**	**0.20**	**0.06–0.66**	**0.009**
Prior lenalidomide	**16/44**	**2.68**	**1.17–6.12**	**0.02**
Maintenance (frontline transplant)	22/44	0.58	0.25–1.35	0.21
Response status after frontline transplant				
≥VGPR vs. <VGPR ≥nCR vs. <nCR	35/44	0.90	0.30–0.27	0.85
	18/44	0.89	0.38–2.11	0.80
Best response during frontline treatment				
≥VGPR vs. <VGPR ≥nCR vs. <nCR	40/44	0.93	0.22–3.99	0.92
	29/44	0.78	0.32–1.89	0.57

*nCR:* near complete remission; *TTP1:* time to progression after frontline transplant; and VGPR: very good partial response. Bold number indicates a significant *p* value.

**Table 3:** Multivariate Cox regression analysis of prognostic impact on progression free survival (PFS) from salvage HDCT/ASCT.

Variable	HR	95% CI	*p*
TTP1	0.74	0.50–1.09	0.13
Prior lines of therapy (>1 vs. 1)	1.80	0.46–7.03	0.40
Response status at time of salvage transplant *	**0.18**	**0.06–0.48**	**0.001**
Maintenance (salvage transplant)	**0.22**	**0.06–0.81**	**0.02**
Prior lenalidomide	2.07	0.78–5.48	0.14

* (≥*VGPR* vs. <*VGPR*); *TTP1:* time to progression after frontline transplant; VGPR: very good partial response. Bold number indicates a significant *p* value.

## Data Availability

The data presented in this study are available on request from the corresponding author.

## Supplementary Materials

The following are available online at https://www.mdpi.com/article/10.3390/cancers13184706/s1: Figure S1: Kaplan-Meier plots of progression-free survival after frontline and salvage HDCT/ASCT according to response, Table S1: Adverse events, antimicrobial management, and transfusions during frontline and salvage HDCT/ASCT.
