# Supplementary material for: Carfilzomib, Lenalidomide, and Dexamethasone Followed by Salvage Autologous Stem Cell Transplant with or without Maintenance for Relapsed or Refractory Multiple Myeloma
Source: Cancers (Basel). 2021 Sep 20;13(18):4706. doi: 10.3390/cancers13184706 (PMC8472377; doi:10.3390/cancers13184706)
Supplement: Supplementary file 1 [file cancers-13-04706-s001.zip › cancers-1350359-supplementary.pdf]

Supplement

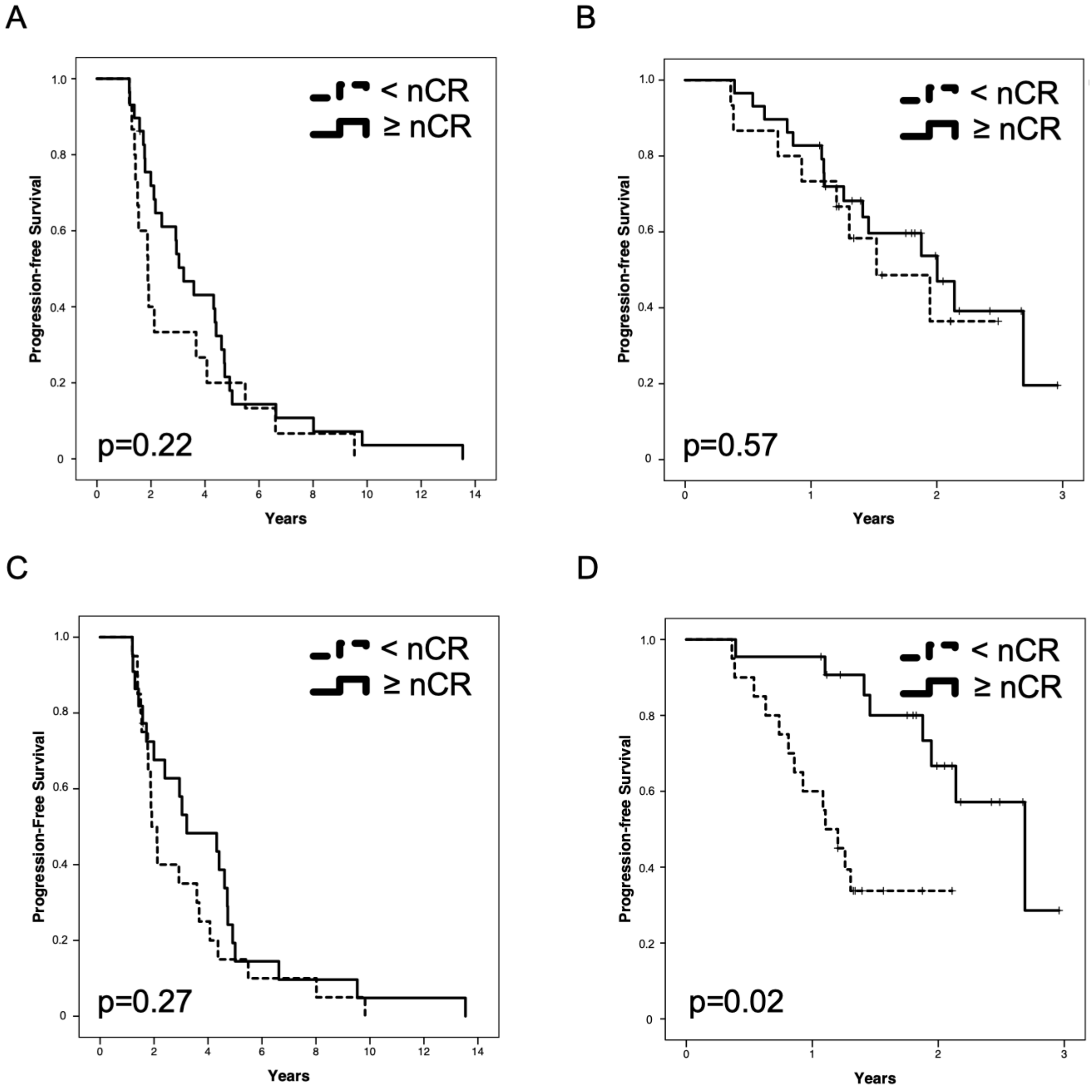

Supplementary Figure S1: Kaplan-Meier plots of progression-free survival after frontline (A, C) and salvage HDCT/ASCT (B, D) according to best response (≥nCR vs. <nCR) during frontline (A, B) and salvage (C, D) treatment. P-values were determined by Cox regression analysis.

Supplementary Table S1: Adverse events, antimicrobial management and transfusions during frontline and salvage HDCT/ASCT Data are n/N (%) or median (range) *covering pseudomonas, §antibiotics with exclusive activity against gram-positive bacteria, &covering aspergillus. *i.v.:* intravenous

Variable	Frontline transplant	Salvage transplant	p
**Infection**	40/43 (93%)	41/44 (93%)	1.0
**Site of infection**			
**Fever of unknown origin**	32/43 (74%)	29/44 (66%)	0.45
**Sepsis**	6/43 (14%)	5/44 (11%)	1.0
**Soft tissue**	1/43 (2%)	2/44 (5%)	1.0
**Respiratory**	1/43 (2%)	3/44 (7%)	0.63
**Gastrointestinal**	0/43	2/44 (5%)	0.5

Urogenital	0/43	0/44	
Blood cultures			
Bacteremia	10/41 (24%)	6/39 (15%)	0.39
Gram-positive	8/41 (20%)	5/39 (13%)	0.29
Gram-negative	2/41 (5%)	2/39 (5%)	1.0
Fungemia	-	1/39 (3%)	1.0
Erythrocyte transfusions	18/44 (41%)	12/44 (27%)	**0.04**
1	-	4/44 (9%)	
2	16/44 (36%)	8/44 (18%)	
>2	2/44 (5%)	-	
Platelet transfusions	24/44	23/44	0.54
1	23/44	19/44	
>1	1/44	4/44	
I.v. antibiotics			
Median Duration [days]	8 (0-18)	8 (0-34)	0.69
Broad-spectrum Beta-lactam*	39/43 (91%)	32/44 (73%)	**0.02**
Carbapenem	22/43 (51%)	19/44 (43%)	0.66
Broad-spectrum Beta-lactam* and/or Carbapenem	39/43 (91%)	34/44 (77%)	0.11
Gram-positive[§]	10/43 (23%)	10/44 (23%)	1.0
Reserve antibiotics	-	-	
Antimycotics[&]	0/43	1/44 (2%)	1.0
Diarrhea			
Frequency	36/43 (84%)	41/44 (93%)	0.13
Median Duration [days]	4.5 (0-17)	8 (0-16)	**<0.001**
Mucositis	41/42 (98%)	39/41 (95%)	0.31
Grade 0	1/42 (2%)	2/41 (5%)	
Grade 1	13/42 (31%)	11/41 (27%)	
Grade 2	15/42 (36%)	9/41 (22%)	
Grade 3	13/42 (31%)	18/42 (43%)	
Stage 4	-	1/41 (2%)	
Pain scale maximum [median]	5 (0-9)	5 (0-8)	0.23
Continuous i.v. antiemetics	-	1/43 (2%)	1.0
Parenteral nutrition	4/44 (9%)	3/44 (7%)	1.0
Continuous i.v. opioids	6/44 (14%)	6/44 (14%)	1.0
Intensive care unit admission	-	2/44 (5%)	0.5
Dialysis	-	-	
Mechanical ventilation	-	1/44 (2%)	1.0
Catecholamines	-	1/44 (2%)	1.0
